# Prioritizing research on over-the-counter (OTC) hearing aids for age-related hearing loss

**DOI:** 10.3389/fragi.2023.1105879

**Published:** 2023-03-23

**Authors:** Vinaya Manchaiah, De Wet Swanepoel, Anu Sharma

**Affiliations:** ^1^ Department of Otolaryngology-Head and Neck Surgery, University of Colorado School of Medicine, Aurora, CO, United States; ^2^ UCHealth Hearing and Balance, University of Colorado Hospital, Aurora, CO, United States; ^3^ Virtual Hearing Lab, Collaborative Initiative Between The University of Colorado and The University of Pretoria, Aurora, CO, United States; ^4^ Department of Speech-Language Pathology and Audiology, University of Pretoria, Pretoria, South Africa; ^5^ Department of Speech and Hearing, Manipal College of Health Professions, Manipal Academy of Higher Education, Manipal, India; ^6^ Ear Science Institute Australia, Perth, WA, Australia; ^7^ Brain and Behavior Laboratory, Department of Speech Language and Hearing Sciences, Institute of Cognitive Science, Center for Neuroscience, University of Colorado Boulder, Boulder, CO, United States

**Keywords:** hearing aids, over-the-counter hearing aids, direct-to-consumer hearing devices, mild-to-moderate hearing loss, healthcare research, age-related hearing loss

## Abstract

Hearing aids are the most commonly used treatment for people with age-related hearing loss, however, hearing aid uptake is low, primarily due to high cost of the device, stigma, and a lack of perceived need. To address accessibility and affordability issues, the U.S. Food and Drug Administration created a new over-the-counter (OTC) hearing aid category. Various types of hearing devices are available for both individuals with hearing loss and for those with normal hearing, as hearing enhancement devices. Hearing aids (i.e., prescription hearing aids, self-fitting OTC hearing aids, and pre-set OTC hearing aids) are regulated by the FDA. The purpose of this article is to (a) provide a summary of existing research on direct-to-consumer (DTC) hearing devices such as Personal Sound Amplification Products (PSAPs) that informs OTC service delivery models; (b) provide an update on existing and ongoing randomized controlled trials on currently marketed OTC hearing aids; and (c) highlight the need for immediate research on OTC hearing aids and service delivery models to inform policy and clinical care. It remains to be seen what effect OTC hearing aids have on improving the uptake of hearing aids by individuals with mild-to-moderate hearing loss. However, there is scant research on all aspects of OTC hearing aids that are currently on the market. We conclude that high quality independent research must be prioritized to supplement evidence provided by the OTC hearing aid manufacturers for regulatory approval purposes.

## 1 Introduction

Aging is the leading cause of hearing loss which affects an estimated 1.5 billion persons globally and age-related hearing loss is one of the most common chronic health conditions affecting nearly one third of the world’s population over 60 years (World Health Organization, 2021). Age-related hearing loss has various physical, cognitive and emotional consequences including structural and functional changes to the brain (Glick and Sharma, 2020; Slade et al., 2020). Hearing aids are the most commonly used treatment for people with hearing loss and the 2020 Lancet Commission on Dementia Prevention, Intervention and Care, identified hearing loss as the leading modifiable, (e.g., through management options such as hearing aids), mid-life risk factor for later development of dementia (Livingston et al., 2020). However, hearing aid uptake is low with only one in four people with hearing loss in high-income countries using hearing aids (Reed et al., 2021). This low uptake has been attributed to several reasons including awareness, high cost of the device, stigma, and a lack of perceived need. To address accessibility and affordability issues with HAs, the Over-the-Counter (OTC) Hearing Aid act passed by the U.S. Congress, 2017 mandated the U.S. Food and Drug Administration (FDA) to release a new category of devices, OTC hearing aids, which consumers can purchase without consulting a licensed hearing healthcare provider. The FDA finalized this decision on 16 August 2022 calling it historic and OTC hearing aids began being sold in the U.S. from 17 October 2022.

**FIGURE 1:**
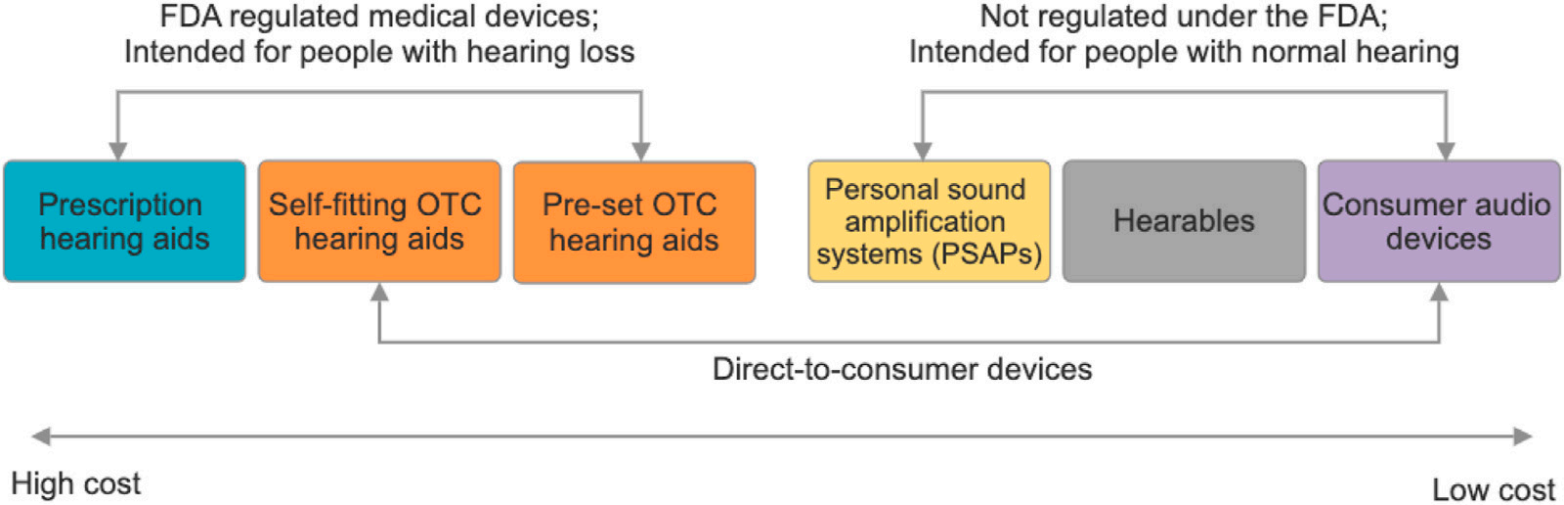
Hearing device categories.

Hearing devices have seen tremendous evolution in the last decade including rapid development in features, functionalities as well as look and feel of the device. Modern hearing aids have many new features such as Bluetooth connectivity, rechargeability and fitness tracking. Interestingly, several devices look more like an earbud rather than a traditional hearing aid. This has been possible due to the convergence of traditional hearing aids, which are medical devices, with consumer audio devices, creating a whole array of hybrid devices such as Personal Sound Amplification Products (PSAPs) and hearables. Currently, there are several hearing devices on the market, of which some are medical devices intended for individuals with hearing loss and regulated by the FDA (i.e., prescription hearing aids, self-fitting OTC hearing aids, pre-set OTC hearing aids), and other devices that serve as hearing enhancement devices for individuals with normal hearing who have an average hearing thresholds of 25 dB or better in frequencies 500 Hz, 1,000 Hz, 2000 and 4,000 Hz (i.e., PSAPs, hearables) or those used mainly for entertainment purpose (i.e., consumer audio devices) (Manchaiah et al., 2023) (Figure 1). Some manufacturers are blurring the lines between these categories and offering sound enhancement and personalization of acoustic output for persons with hearing loss using smartphone-based earphones with an accompanying smartphone app (Lin et al., 2022). Moreover, studies have documented that people with hearing loss tend to use devices such as PSAPs and hearables which are meant to be for people with normal hearing (Kochkin, 2010; Manchaiah et al., 2019).

Some of these devices have been available to consumers through direct-to-consumer (DTC) channels for several years (i.e., PSAPs, hearables) including a category of DTC online hearing aids. The OTC hearing aid category has now superseded the DTC online hearing aid category with devices becoming available in-store and online to consumers in the United States starting 17 October 2022 following the historic ruling of the U.S. Food and Drug Administration (Food and Drug Administration, 2022). The OTC hearing aids are for adults with perceived mild-to-moderate hearing loss which generally tend to be individuals with age-related hearing loss. Although consumers who purchase these devices without consultation with hearing healthcare professionals (i.e., audiologists, otolaryngologists) may have some risk of not having the opportunity to identify possible medical conditions (e.g., middle ear disorders) resulting in hearing loss (Hoff et al., 2020), consensus expert opinion is that the benefits of OTC hearing aids will outweigh the limitations (Warren, & Grassley, 2017).

**FIGURE 2:**
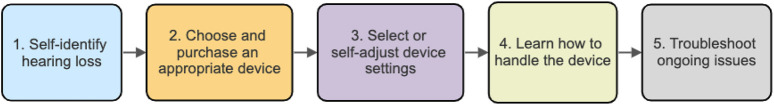
Pre-requisites for successful use of OTC hearing aids along the consumer journey.

There are some important pre-requisites for successful use of OTC hearing aids to achieve optimal benefit and satisfaction as illustrated in Figure 2. First, consumers must self-identify their hearing loss and also ensure that they have no ear disorders. This is important as OTC hearing aids are intended for individuals with self-perceived mild to-moderate-hearing loss. Hence, over- or under-estimation of hearing loss or not recognizing ear disorders may pose a potential barrier to optimal benefits. Second, users have to consider several device options on the market, choose and purchase an appropriate device *via* channels such as supermarkets, pharmacies, consumer electronic stores or online. Third, users may have to select one of the pre-set programs or self-fit the hearing aid *via* an accompanying smartphone app. Fourth, users have to self-learn handling skills such as putting on the hearing aid appropriately, charging, cleaning, etc. Finally, users must monitor on-going issues with the device (e.g., connectivity with the smartphone app, no sound due to earwax blocking the speaker of the device) and troubleshoot them as necessary. OTC hearing aids do come with instruction manuals and/or step-by-step help in the smartphone app. Moreover, users may also have remote customer support by a technician and/or remote clinical support by hearing healthcare professionals. Nevertheless, these are still pre-requisites that users need to be able to comply with to obtain optimal benefits. For these reasons, both consumers as well as the licensed hearing care professionals/providers (HCPs) who are assisting the consumers must consider aspects related to the (a) device, (b) service delivery model and (c) user when deliberating on the appropriateness of OTC hearing aids for specific persons.

In this article, we aim to 1) provide a summary of existing research on DTC hearing devices that informs OTC service delivery models; 2) provide an update on existing and ongoing randomized controlled trials (RCTs) on currently marketed OTC hearing aids based on the clinical trials registration; and 3) highlight the need for immediate research on OTC hearing aids and service delivery models to inform policy and clinical care.

## 2 Discussion

### 2.1 Previous research on DTC hearing devices and service delivery models

Much of the existing research on this area was conducted pre-2017 when the OTC hearing aid category did not exist in the United States. As the hearing devices (i.e., PSAPs, hearables, direct-mail hearing aids) used in the research discussed below were available to consumers *via* DTC channel, we can call them as DTC hearing devices. Nevertheless, a few systematic reviews on consumer hearing devices suggested that the available literature can be grouped into three key themes focusing on 1) acoustic quality of hearing devices, 2) consumer surveys, and 3) clinical trials as discussed below (Manchaiah et al., 2017; Maidment et al., 2018; Tran & Manchaiah, 2018; Chen et al., 2022). First, a series of studies examined electroacoustic characteristics in the test box (e.g., frequency response, distortion, equivalent input noise) of DTC hearing devices such as PSAPs and hearables which showed mixed results. Some studies showed that these devices were of very poor acoustic quality (Callaway & Punch, 2008; Chan and McPherson, 2015), whereas other studies concluded that some of these devices have appropriate acoustic characteristics for people with hearing loss (Reed et al., 2017). These results highlight the importance of quality of acoustic output in device selection. It is noteworthy that some of these studies point to the fact that higher priced devices generally have better acoustic quality (Almufarrij et al., 2019). It is also important to note that most of the evaluated devices are not currently offered as FDA-regulated OTC devices. Second, a few large-scale consumer surveys on DTC hearing aid users in the United States (Kochkin, 2010) and Japan (EHIMA, 2022) show that the benefit and satisfaction reported by users of devices such as PSAPs and direct-mail hearing aids is generally much lower when compared to users of prescription hearing aids fitted by HCPs. The reason for this can be attributed to the poor quality of DTC devices available a decade ago, as well as users may not have met one of the five pre-requisites discussed above. Finally, the third group of studies included clinical trials focused on the outcomes of DTC hearing devices (Maidment et al., 2018; Tran & Manchaiah, 2018; Chen et al., 2022). These studies generally showed positive outcomes in self-reported hearing aid benefit and satisfaction measures [e.g., Abbreviated Profile of Hearing Aid Benefit (APHAB)] as well as in behavioral measures (e.g., speech in quiet, speech in noise). However, the main criticism of these studies is that they generally used single-group pretest-posttest study designs without a control group (e.g., Sacco et al., 2016; Mamo et al., 2017).

In another study, Humes et al. (2017) performed a three-arm double-blind placebo-controlled trial comparing a gold standard audiologist fitted group with a consumer decides self-fit group and a placebo group (*n* = 154 across all groups). Participants from all three groups used prescription hearing aids, although the self-fit group used a device with pre-set programs and the placebo group used a device with no functional gain. The study results showed that the self-fit group presented with only slightly poorer outcomes in self-reported and behavioral measures when compared to an audiologist-fitted group demonstrating the efficacy of the OTC service delivery model. In a follow-up study, Humes et al. (2019) further examined the consumer decides self-fit model with less front-end screening in a double-blind clinical trial (*n* = 40). Participants were asked to choose one of the pre-programed hearing aids (like a pre-set OTC hearing aid) one of which included a placebo device with functional gain. The outcomes of the two groups with pre-programmed hearing aids with gain were comparable and were superior to the placebo group. The study also highlighted that the presence of red-flag conditions (e.g., cerumen) did not impact the purchase decision of the users raising some concerns about consumers ability to self-identify their candidacy for OTC hearing aids.

All the studies discussed above used either early generation DTC hearing devices (Manchaiah et al., 2017; Tran & Manchaiah, 2018) or prescription hearing aids with limited features to simulate an OTC hearing aid (Humes et al., 2017; Humes et al., 2019). Moreover, they focused on either the device or the service delivery model which limits the ecological validity and generalizability. Overall, the key takeaway from these studies is that if users choose an appropriate device, then they are likely to have some measurable benefit from using them.

### 2.2 Existing and ongoing research on OTC hearing aids and service delivery models

Table 1 presents a summary of completed as well as on-going RCTs studies examining OTC hearing aids and/or the service delivery models. Of these, only two studies have been published in a peer reviewed journal (Sabin et al., 2020; De Sousa et al., 2023) and two other studies are marked as complete in the clinical trials registry. It appears that most of these RCTs (4 of the 8 listed in Table 1) are industry sponsored studies examining self-fitting algorithms or process when compared to audiologist-fitted devices for regulatory approval from the FDA. Two of the ongoing studies that are funded by a federal agency (National Institute on Deafness and Other Communication Disorders; NIDCD) and a non-profit organization (i.e., Patient-Centered Outcomes Research Institute; PCORI) seem to have a large sample size (*n* = 240–591) and aim to investigate service delivery models. In most of the studies, OTC hearing aids that are currently on the market or likely to come to the market in the near future are being investigated which increases their ecological validity. All of the studies include self-reported and/or behavioral measures as primary and secondary outcome measures. Unlike blinded RCTs (e.g., Humes et al., 2017, 2019), these studies use devices that are currently on the market with the existing branding information. There may be some placebo effects that could potentially impact outcomes of both self-reported and behavioral outcomes (Dawes et al., 2013). This highlights a need for studies that also include more objective outcomes like electrophysiological markers to examine non-subjective benefits of these devices and associated service delivery models (Glick & Sharma, 2020).

**TABLE 1:** Controlled trials (completed and on-going) on OTC hearing aids and service delivery models.

Study sponsor or principal investigator	Status	Funding source	Arms and design (n)	Hearing device categories	Hearing aid brand/model	Outcome domains studied
Sabin et al. (2020); Ear Machine LLC transferred to Bose Corporation	Complete	Federal (NIDCD)	2 arms parallel assignment: AF vs. SF (*n* = 75)	SF OTC hearing aid and AF version of the same device	Bose sound control	Self-reported, Behavioral
GN Hearing A/S	Complete*	Industry	2 arms cross-over design: AF vs. SF (*n* = 40)	SF OTC hearing aid and AF version of the same device	Jabra Enhance Plus	Self-reported, Behavioral
Sousa et al. (2023); hearX group	Complete	Industry	2 arms parallel assignment: AF vs. SF (*n* = 68)	SF OTC hearing aid and AF version of the same device	Lexie Lumen	Self-reported, Behavioral
Yu-Hsiang Wu	Ongoing	Federal (NIDCD)	3 arms parallel assignment: AF vs. SF vs. Hybrid (*n* = 240)	Prescription HAs in AF; Pre-set OTC in other two groups	Unknown	Self-reported, Behavioral
Northwestern University	Ongoing	Independent non-profit (PCORI)	3 arms parallel assignment: AF vs. SF-1 (consumer decides) vs. SF-2 (efficient fitting) (*n* = 591)	Prescription HAs in all three groups	Unknown	Self-reported, Behavioral
University of Minnesota	Ongoing	University of Minnesota	2 arms cross-over design: AF vs. SF (*n* = 40)	SF OTC hearing aid and AF version of the same device	Eargo	Self-reported, Behavioral
Whisper AI	Ongoing	Industry	2 arms cross-over design: AF followed by SF vs. placebo (*n* = 80)	SF OTC hearing aid and AF version of the same device	Whisper AI	Self-reported, Behavioral
Starkey Laboratories Inc.	Complete*	Industry	2 arms cross-over design: AF vs. SF (*n* = 40)	SF OTC hearing aid and AF version of the same device	Start Hearing One	Self-reported, Behavioral

; * Note: Unpublished study details were retrieved from the clinicaltrials.gov registry, Marked as complete in the Clinical trials registry but the results are not published yet; SF, self-fitting; AF, audiologist-fitted; HAs, hearing aids; NIDCD, national institute on deafness and other communication disorders; PCORI, patient-centered outcomes research institute.

### 2.3 Outstanding research questions about OTC hearing aids and service delivery models

There is an immediate need for research on all aspects of OTC hearing aids including hearing device characteristics, service delivery models, the user and the complex interaction between these three domains. The following are some aspects that we think are highly relevant and timely to inform hearing healthcare policy, clinical care, industry decisions and increase much-needed hearing aid uptake. The key question during the last decade was whether OTC hearing aids provide measurable benefit to users. This question continues to be important but since devices are already available on the marketplace through a regulatory framework (requiring FDA non-inferiority trials for self-fitting OTC hearing aids), other pressing questions should be prioritized. Important questions should consider for whom OTC devices and respective service delivery models work and what the predictors of success are. We outline some specific questions below:

Hearing devices related:

⁃ What is the range in electroacoustic characteristics of OTC hearing aids that are currently on the market and how many of them are appropriate for individuals with mild-to-moderate hearing loss?
⁃ How effective are self-fitting algorithms in personalizing hearing aid gain for individual users when compared to the gold standard prescription targets (NAL-NL2) based on pure tone audiometry thresholds? Also, is there a difference in different self-fitting methods (e.g., *in-situ* audiometry-based fitting vs. direct methods)?
⁃ Is there a difference in outcomes between pre-set vs. self-fitting OTC hearing aids?
⁃ What are the outcomes of OTC hearing aids that are currently available when compared to prescription hearing aids (e.g., Swanepoel et al., 2023) as well as other type of DTC hearing devices such as PSAPs or hearables?
⁃ Is there incremental benefit and satisfaction from OTC hearing aid users from incremental technology?

Service delivery model related:

⁃ What are the contextual facilitators and barriers to implementation of OTC service delivery models in different settings according to stakeholders such as users, HCPs, patient organizations, managers of the health systems, companies manufacturing and distributing OTC hearing aids as well as potential payers such as insurance companies?
⁃ What role do HCPs play in facilitating the journey of users of OTC hearing aids? What guidance, additional training and support would HCPs need if they include OTCs in their practice.
⁃ How cost-effective are OTC hearing aids from the payer as well as provider perspective?
⁃ What effect do OTC hearing aids have on hearing aid market in terms of improving uptake rates, reducing hearing aid costs, improving access to people with low-incomes and ethnic minorities, and in improving the features and functionalities of all categories of devices?
⁃ Are OTC hearing aids, including the use of consumer brands, improving the traditional stigma surrounding hearing loss and hearing aids.
⁃ What outcome measures are best suited to evaluated OTC hearing aids (e.g., behavioral, subjective, cognitive, objective brain-based)?
⁃ Are there measures [e.g., Digits-In-Noise (DIN) test] that can be administered over the internet (web or mobile phone) that can predict who will benefit from OTC hearing aids as well can be used as an outcome measure?

User related:

⁃ Are there specific users based on their biographical, demographic, and audiological variables who are more likely to seek OTC hearing aids and successfully navigate the OTC service delivery model in terms of key prerequisites?
⁃ How and where will consumers find OTC hearing aid models? How will they make decisions on which device to purchase?
⁃ How will users determine whether they are benefitting from OTC hearing aids or whether they should return or exchange the hearing aids during the federally mandated trial period? It is possible to design a self-testing method that consumers can use for this purpose?
⁃ Will there be a difference with respect to when and where users are more likely to wear OTC vs. prescription hearing aid? For example, are OTC hearing aids more likely to be used situationally while prescription hearing aids used daily? How does the duration of hearing aid use impact core outcomes?
⁃ What is the cost-benefit ratio and for which users when it comes to lower versus higher cost OTC hearing aids and which features, and functionalities are most relevant?
⁃ Can customized or personalized educational programs increase user uptake and motivation, supplement self-fitting and management of OTC hearing aids and enhance outcomes (e.g., Ferguson et al., 2021)?

## 3 Conclusion

OTC hearing aids have opened a new service-delivery avenue for hearing care with many potential consumer benefits especially for those with age-related hearing loss. The limited available research on OTC hearing aids currently on the market emphasizes the need for a stronger evidence-base to support the efficacy of these devices and their service-delivery models. In this article, we propose several questions regarding OTC hearing aids as well as service delivery models that need to be answered rigorously and urgently to inform hearing healthcare policy and clinical care. High quality independent research is important to supplement the evidence that is currently being provided by the OTC hearing aid manufacturers for regulatory approval. Moreover, Patient and Public Involvement (PPI) or Consumer and Community Involvement (CCI) should be considered in shaping future research priorities (Dawes et al., 2022).

## Data Availability

The original contributions presented in the study are included in the article/Supplementary Material, further inquiries can be directed to the corresponding author.
